# Comparison of Single-Trait and Multi-Trait Genome-Wide Association Models and Inclusion of Correlated Traits in the Dissection of the Genetic Architecture of a Complex Trait in a Breeding Program

**DOI:** 10.3389/fpls.2021.772907

**Published:** 2022-01-28

**Authors:** Lance F. Merrick, Adrienne B. Burke, Zhiwu Zhang, Arron H. Carter

**Affiliations:** Department of Crop and Soil Sciences, Washington State University, Pullman, WA, United States

**Keywords:** covariates, single-locus model, multi-locus model, reduced height alleles, pleiotropic effects, seedling emergence, coleoptile length

## Abstract

Unknown genetic architecture makes it difficult to characterize the genetic basis of traits and associated molecular markers because of the complexity of small effect quantitative trait loci (QTLs), environmental effects, and difficulty in phenotyping. Seedling emergence of wheat (*Triticum aestivum* L.) from deep planting, has a poorly understood genetic architecture, is a vital factor affecting stand establishment and grain yield, and is historically correlated with coleoptile length. This study aimed to dissect the genetic architecture of seedling emergence while accounting for correlated traits using one multi-trait genome-wide association study (MT-GWAS) model and three single-trait GWAS (ST-GWAS) models. The ST-GWAS models included one single-locus model [mixed-linear model (MLM)] and two multi-locus models [fixed and random model circulating probability unification (FarmCPU) and Bayesian information and linkage-disequilibrium iteratively nested keyway (BLINK)]. We conducted GWAS using two populations. The first population consisted of 473 varieties from a diverse association mapping panel phenotyped from 2015 to 2019. The second population consisted of 279 breeding lines phenotyped in 2015 in Lind, WA, with 40,368 markers. We also compared the inclusion of coleoptile length and markers associated with reduced height as covariates in our ST-GWAS models. ST-GWAS found 107 significant markers across 19 chromosomes, while MT-GWAS found 82 significant markers across 14 chromosomes. The FarmCPU and BLINK models, including covariates, were able to identify many small effect markers while identifying large effect markers on chromosome 5A. By using multi-locus model breeding, programs can uncover the complex nature of traits to help identify candidate genes and the underlying architecture of a trait, such as seedling emergence.

## Introduction

Complex traits are controlled by many quantitative trait loci (QTLs) and are influenced by environmental conditions (Bernardo, 2020). Challenges due to complexity, small effect QTLs, and difficulty in phenotyping can make it difficult to characterize the genetic basis of traits and associated molecular markers, especially in biparental populations. Linkage mapping for complex traits can often result in inconsistent estimated QTL effects (Bernardo, 2020; Tibbs Cortes et al., 2021). Unfortunately, many of these complex traits are essential for selection in plant breeding programs, typically being associated with yield potential, end-use quality, and certain biotic and abiotic stress types of tolerance. Therefore, there is a need to increase the knowledge of the inheritance and genetic architecture of these complex traits (Bernardo, 2020).

In recent years, the use of genome-wide association studies (GWASs) has enabled the discovery of QTLs in a collection of diverse populations or diversity panels rather than using a mapping population (Lander and Schork, 1994). GWAS can be performed to dissect the genetic architecture of a trait by exploiting all historical recombination events in the population and allow for the ability to understand the genetic basis by identifying the associations between genetic markers and phenotypes (Lipka et al., 2015). Not only are complex traits influenced by the environment and multiple QTLs, but they also interact with correlated traits that result in a complex genetic architecture. Using heritable covariates and correlated secondary traits can help account for confounding factors that bias marker effects and improve the power of a GWAS model (Aschard et al., 2015). Additionally, using population structure or genetic relatedness controls *p*-value inflation for each marker and reduces false positives (Tibbs Cortes et al., 2021).

Statistical models have been developed for GWAS to distinguish real associations from false positives caused by population structure and linkage disequilibrium (LD). Some of the first GWAS models, such as general linear models and mixed-linear models (MLMs), were single-locus, single-trait GWAS (ST-GWAS) models created to implement the covariates along with kinship matrices (Yu et al., 2006). However, these simple models resulted in false negatives caused by weakened associations in order to control inflation of *p*-values due to population structure (Liu et al., 2016). MLMs were improved upon by compressed MLMs (CMLMs), which cluster individuals and use them as random effects rather than individual genotypic effects (Zhang et al., 2010). CMLMs were further improved by using pseudo quantitative trait nucleotides (QTNs) to derive kinship instead of all the genetic markers. The settlement of MLM under progressively exclusive relationship (SUPER) model sorts markers by association and then combines them into bins with the most significant marker designated as pseudo QTNs and then used to derive a reduced kinship matrix (Wang et al., 2014). These methods improved computational efficiency and statistical power over MLMs because of weakening real associations when controlling the *p*-value inflation while accounting for population structure (Liu et al., 2016). Compared to single-locus models, newer multi-locus models were then developed to test multiple markers simultaneously (Liu et al., 2016). These multi-locus GWAS models, such as FarmCPU and BLINK, allow for the evaluation of big datasets while also reducing false positives and negatives (Huang et al., 2019). FarmCPU bins markers and fits them as cofactors to control false positives for testing the rest of the markers in a fixed-effect model, and then a random effect model is used to select the associated markers. BLINK eliminates the FarmCPU assumption that causal genes are evenly distributed across the genome, which improves speed, because of the optimization of bin size and number no longer being required (Huang et al., 2019). Additionally, multi-trait GWAS (MT-GWAS) can be used to analyze multiple traits simultaneously. MT-GWAS methods were developed to increase statistical power and identify pleiotropic loci (Porter and O’Reilly, 2017). Correlations and pleiotropy can be used to increase power compared to ST-GWAS (Galesloot et al., 2014). MT-GWAS methods display increase in power even when traits display negative correlation, when only one of the traits is associated with the loci, or when genetic correlations among traits are weak (Galesloot et al., 2014). However, because of the intricate and pleiotropic nature of quantitative traits, there is no best model for all situations, and it is recommended to compare models to dissect the unique genetic architecture of complex traits (Tibbs Cortes et al., 2021).

In Washington state, seedling emergence of deep-sown winter wheat is a complex trait affected by many factors and is dependent on the environment to display variation (Schillinger et al., 1998; Lutcher et al., 2019). Seedling emergence is dependent on deep sowing at depths of 10–15 cm when precipitation is below 150–300 mm annually (Schillinger et al., 1998; Mohan et al., 2013). Under these limited moisture conditions, a winter-wheat summer fallow rotation is employed. Additionally, rod-weeding is used in the fallow year to control weeds as well as limit evapotranspiration, thereby conserving what moisture may be in the soil at depths beneath the rod-weeding implement. In deep-sowing practices, fast-emerging cultivars are required to emerge before precipitation events create soil crusting that can dramatically decrease emergence. Wheat seedlings have decreased emergence when they cannot penetrate the soil surface because of crusting, due to the inability to germinate under dry soil conditions, or short coleoptiles. Seedling emergence and, therefore, stand establishment are vital factors affecting grain yield in these growing regions in Washington state and can reduce grain yields by 35–40% (Schillinger et al., 1998).

Previous studies have shown a significant positive relationship between coleoptile length and seedling emergence (Allan et al., 1962; Sunderman, 1964; Chastain et al., 1995; Schillinger et al., 1998; Botwright et al., 2001; Schillinger, 2011). The reduced height (Rht) genes *Rht-B1b* and *Rht-D1b* are mutant alleles that cause the semi-dwarfing stature of wheat (Vogel et al., 1956; Allan et al., 1962). Dwarfing genes are responsible for short stature and have pleiotropic effects that include gibberellin insensitivity, coleoptile length, yield, protein content, and disease resistance (Gale and Youssefian, 1983; Allan, 1989). Semi-dwarf wheat cultivars have improved resistance to lodging and grain yield but reduced coleoptile length by one-half to three-fourths of the standard varieties at the time of their development (Allan et al., 1961; Allan, 1989; Mohan et al., 2013). The reduced coleoptile length was due to decreased gibberellic acid response, which reduced cell size and elongation (Allan et al., 1959). Historically, when crusting was present, or other unfavorable conditions, the shorter coleoptiles of semi-dwarf cultivars resulted in poor stand establishment and yield potential (Rebetzke et al., 1999).

Recently, after 60 years of breeding, emergence in modern varieties was shown to have a reduced correlation between emergence and coleoptile length (Mohan et al., 2013). Coleoptile length only accounted for 28% of the variability for seedling emergence, and some lines with short coleoptiles had the best emergence rating. The remaining variability is attributed to many factors that affect seedling emergence, leading to a complex system resulting in stand establishment. As stated previously, the two main scenarios that affect seedling emergence include adequate seed-zone water potential and the occurrence of surface soil crust that prevents penetration (Schillinger, 2011). Successful seedling emergence is dependent on the force exerted by the first leaf. The first leaf protrudes through the coleoptile and emerges around 10–12 days after planting. During this time, the first leaf can be prone to buckling before it emerges, which can be affected by coleoptile diameter, speed of emergence, emergence force, and lifting capacity of the first leaf, along with the associated coleoptile length (Arndt, 1965; Schillinger et al., 2017; Lutcher et al., 2019). Adequate seed zone water potential is associated with seed germination and impacts the speed of emergence because of water availability (Evans and Etherington, 1990; Pill, 1995). These studies showed that the genetic basis of seedling emergence is a complex trait not solely controlled by genes for any one factor and results in a poorly understood genetic architecture that is dependent on the environment to display variation (Schillinger et al., 2017; Lutcher et al., 2019). This study presents research to assess the genetic architecture of a complex trait by (1) comparing ST-GWAS and MT-GWAS models for correlated traits and (2) assessing the inclusion of fixed effects to improve the power to explore the genetic architecture of seedling emergence.

## Materials and Methods

### Phenotypic Data

Seedling emergence notes were taken on research plots under low annual precipitation (∼150 mm annual precipitation) at the Washington State University Dryland Research Center in Lind, WA (47.001552, −118.565556). The plots were planted using a custom-built deep-furrow planting system to a depth of between 10 and 15 cm, depending on moisture variation among years. The plots were planted 1.5 m wide and 6.1 m long with 31 cm row spacing at a density of 120 seed per square meter. Emergence notes were taken on two populations within the breeding program. The diverse association mapping panel (DP) represents a diverse panel of inbred breeding lines (BLs) from Pacific Northwest breeding programs not selected exclusively in deep-furrow trials. In contrast, the second population is composed of F_3:5_ BLs and represents a population of closely related lines from a single breeding program composed of pedigrees that have been selected for emergence over previous generations and is a part of the WSU breeding program. The two populations were used to compare GWAS models. The DP was used as the primary population for genetic dissection, and the BL population used as the validating population. The DP was evaluated in 2015, 2017, 2018, and 2019 in Lind, WA (Table 1). The BLs were planted using an unreplicated augmented design that was evaluated in 2015 for emergence (Table 1). In 2016, no data were collected for the DP because of significant soil crusting that was severe enough to impede the seedling emergence of all lines.

**TABLE 1:** Populations for seedling emergence screened in unreplicated trials under moisture stress in Lind, WA from 2015 to 2019.

Location	Trial	Year	Individuals
Lind	DP*	2015	473
Lind	DP	2017	473
Lind	DP	2018	473
Lind	DP	2019	473
Lind	F_3:5_	2015	276

*DP: quality association mapping diversity panel.

Seedling emergence was visually assessed and recorded as a percentage of the total plot that emerged 6 weeks after planting for each trial. Table 1 summarizes location, population, year, and the number of genotyped individuals. The emergence issue for each trial was attributed to moisture stress. Coleoptile length was also measured for the DP in 2014 and in two replicates in 2016 under greenhouse conditions. Coleoptile length was recorded to the nearest millimeter according to Murphy et al. (2008).

### Phenotypic Adjustments

Adjusted means from the emergence data collected in the unreplicated trials were adjusted using residuals calculated for the unreplicated lines in individual environments and across environments using the modified augmented complete block design (ACBD) model (Federer, 1956; Goldman, 2019). The adjustments were made following the method implemented in Merrick and Carter (2021), with the full model in a single model as follows:


(1) $${\text{Y}}_{\mathrm{ij}}=\mu +{\text{Block}}_{i}+{\text{Check}}_{j}+\varepsilon_{\mathrm{ij}},$$

where ***Y_ij_*** is the phenotypic value for the trait of interest of the *i*-th block and *j*-th check (*i* = 1,…,I, *j* = 1,…,J); *μ* is the mean effect; ***Block_i_*** is the fixed effect of the *i*-th block; ***Check_j_*** is the fixed effect of the *j*-th replicated check cultivar; and ***ε_ij_*** are residual errors with a random normal distribution of $\varepsilon ∼N\left(0,\sigma_{\varepsilon }^{2}\right)$. For adjusted means across environments, the model is as follows:


(2) $$\begin{array}{c} {\text{Y}}_{\mathrm{ijk}}=\mu +{\text{Block}}_{i}+{\text{Check}}_{j}+{\text{Env}}_{k}+{\text{Block}}_{i}:{\text{Env}}_{k}\\ +{\text{Check}}_{j}:{\text{Env}}_{k}+\varepsilon_{\mathrm{ijk}} \end{array}$$

where ***Y_ij_*** is the phenotypic value for the trait of interest of the *i*-th block and *j*-th check in the *k*-th environment (*i* = 1,…,I, *j* = 1,…,J, k = 1,…,K); *μ* is the mean effect; ***Block_i_*** is the fixed effect of the *i*-th block; ***Check_j_*** is the fixed effect of the *j*-th replicated check cultivar; ***Env_k_*** is the fixed effect of the *k*-th environment; and ***ε_ijk_*** are residual errors with a random normal distribution of $\varepsilon ∼N\left(0,\sigma_{\varepsilon }^{2}\right)$.

Best linear unbiased predictors (BLUPs) for heritability were calculated for each trial and across trials using a mixed linear model for the full augmented randomized complete block design in a single environment and is as follows:


(3) $${\text{Y}}_{\mathrm{ijk}}=\mu +{\text{Block}}_{i}+{\text{Check}}_{j}+{\text{Gen}}_{l\left(j\right)}+\varepsilon_{\mathrm{ijk}}$$

where ***Y_ij_*** is the phenotypic value for the trait of interest of the *l*-th genotype nested in the *j*-th check in the *i*-th block (*i* = 1,…,I, *j* = 1,…,J, *k* = 1,…,K); *μ* is the mean effect; ***Block_i_*** is the random effect of the *i*-th block with the distribution $\text{Block}∼N\left(0,\sigma_{Block}^{2}\right)$; *Check*_*j*_ is the fixed effect of the *j*-th replicated check cultivar; ***Gen***_***k***(***j***)_ is the genotype *k* in the *j*-th check with the distribution $\text{Gen}∼N\left(0,\sigma_{Gen}^{2}\right)$; and ***ε_ij_*** are residual errors with a random normal distribution of $\varepsilon ∼N\left(0,\sigma_{\varepsilon }^{2}\right)$. For the full model (3) across environments is as follows:


(4) $$\begin{array}{c} {\text{Y}}_{\mathrm{ijkl}}=\mu +{\text{Block}}_{i}+{\text{Check}}_{j}+{\text{Gen}}_{l\left(j\right)}+{\text{Env}}_{k}+{\text{Block}}_{i}:{\text{Env}}_{k}\\ +{\text{Check}}_{j}:{\text{Env}}_{k}+{\text{Gen}}_{l\left(j\right)}:{\text{Env}}_{k}+\varepsilon_{\mathrm{ijkl}} \end{array}$$

where ***Y_ijkl_*** is the phenotypic value for the trait of interest of the *l*-th genotype nested in the *j*-th *Check* of the *i*-th *block* and *k*-th environment (*i* = 1,…,I, *j* = 1,…,J, *k* = 1,…,K, *l* = 1,…,L); *μ* is the mean effect; ***Block_i_*** is the random effect of the *i*-th block with the distribution $\text{Block}∼N\left(0,\sigma_{Block}^{2}\right)$; ***Check_j_*** is the fixed effect of the jth replicated check cultivar; ***Gen***_***l***(***j***)_ is the random effect of the genotype *l* in the *j*-th check with the distribution $\text{Gen}∼N\left(0,\sigma_{Gen}^{2}\right)$; ***Env_k_*** is the random effect of the *k*-th environment with the distribution $\text{Env}∼N\left(0,\sigma_{Env}^{2}\right)$; and ***ε_ijkl_*** are residual errors with a random normal distribution of $\varepsilon ∼N\left(0,\sigma_{\varepsilon }^{2}\right)$. Heritability on a genotype-difference basis for broad-sense heritability was calculated using the variance components from models 3 and 4 implemented in Merrick and Carter (2021) and using BLUPs for both individual environments and across environments using the formula from Cullis et al. (2006):


(5) $$H_{Cullis}^{2}=1-\frac{{\bar{v}}_{△..}^{BLUP}}{2\sigma_{g}^{2}}$$

where $\sigma_{g}^{2}$ and $\bar{V}$^BLUP^ are the genotype variance and mean-variance, respectively, of a difference between two BLUPs for the genotypic effect BLUPs (Schmidt et al., 2019). Trial evaluation and significant differences were evaluated using the coefficient of variation, and by analysis of variance (ANOVA) in individual and across trials using BLUP models 3 and 4. BLUPs for coleoptile length were calculated across trials using a mixed linear model as follows:


(6) $${\text{Y}}_{\mathrm{klm}}={\text{Gen}}_{l}+{\text{Env}}_{k}+{\text{Rep}}_{m\left(k\right)}{\text{Gen}}_{l}:{\text{Env}}_{k}+\varepsilon_{\mathrm{klm}}$$

where ***Y_klm_*** is the trait of interest of the *l*-th genotype in the *k*-th environment of the *m*-th replication (*k* = 1,…,K, *l* = 1,…,L, *m* = 1,…,M); ***Gen***_***l***(***j***)_ is the random effect of the *l*-th genotype with $\text{Gen}∼N\left(0,\sigma_{Gen}^{2}\right)$; ***Env_k_*** is the random effect of the *k*-th environment with $\text{Env}∼N\left(0,\sigma_{Env}^{2}\right)$; ***Rep***_***m***(***k***)_ is the random effect of the replication *m* in the *k*-th environment; and ***ε_ijkl_*** are residual errors with a random normal of distribution of $\varepsilon ∼N\left(0,\sigma_{\varepsilon }^{2}\right)$.

Adjusted means for coleoptile length were calculated across trials using a linear model as follows:


(7) $${\text{Y}}_{\mathrm{lm}}={\text{Env}}_{k}+{\text{Rep}}_{m\left(k\right)}+\varepsilon_{\mathrm{lm}}$$

where ***Y_lm_*** is the phenotypic value for the trait of interest of the *k*-th environment of the *m*-th replication (*l* = 1,…,L, *m* = 1,…,M); ***Env_k_*** is the fixed effect of the *k*-th environment; ***Rep***_***m***(***k***)_ is the fixed effect of the replication *m* in the *k*-th environment; and ***ε_ijk_*** are residual errors with a random normal distribution of $\varepsilon ∼N\left(0,\sigma_{\varepsilon }^{2}\right)$. Means were adjusted for coleoptile length following the method for models 1 and 2.

Phenotypic correlations were conducted between seedling emergence in the DP across years along with coleoptile length. Due to the unreplicated nature of the seedling emergence phenotypes, genetic correlations between seedling emergence in the DP and coleoptile length was calculated using the R package “sommer” with the multivariate Newton-Raphson algorithm used for multiple random effects and covariance structures using the multivariate model in Covarrubias-Pazaran (2018):


(8) $$\left[\begin{array}{c} {\text{Y}}_{A}\\ {\text{Y}}_{B} \end{array}\right]=\left[\begin{array}{cc} {\text{X}}_{A} & 0\\ 0 & {\text{X}}_{B} \end{array}\right]\left[\begin{array}{c} \beta_{A}\\ \beta_{B} \end{array}\right]+\left[\begin{array}{cc} {\text{Z}}_{A} & 0\\ 0 & {\text{Z}}_{B} \end{array}\right]\left[\begin{array}{c} {\text{u}}_{A}\\ {\text{u}}_{B} \end{array}\right]+\left[\begin{array}{c} \varepsilon_{A}\\ \varepsilon_{B} \end{array}\right]$$

where ***Y_A_*** and ***Y_B_*** are vectors (n × 1; n = number of lines) of trait-adjusted means for emergence using models 1 and 2, and adjusted means for coleoptile length using model 7, respectively. β_***i***_ is a vector [p × 1; p = three principal components (PCs)] of fixed effects of PCs, **u_*i*_** is a vector (1 × m; m = number of markers) of random effects for individuals with $\text{u}∼N\left(0,\sigma_{\text{u}}^{2}\right)$, and ***ε_i_*** is a vector (n × 1; n = number of lines) of residuals with $\varepsilon ∼N\left(0,\sigma_{\varepsilon }^{2}\right)$ for each trait (I = A…B). **X** and **Z** are incidence matrices for fixed effects (n × p; n = number of lines, p = three PCs) and random genetic effects (n × m; n = number of lines, m = number of markers), respectively, for each trait. The distribution of the multivariate response and phenotypic variance-covariance **V** following the models in Covarrubias-Pazaran (2018) are:


(9) $$\begin{array}{c} \text{Y}=\text{X}\beta +\text{Zu}+\varepsilon \\ \text{Y}∼MVN\left(\text{X}\beta ,V\right)\\ \text{Y}=\left[\begin{array}{c} Y_{A}\\ Y_{B} \end{array}\right]\\ X=\left[\begin{array}{ccc} X_{A} & 0 & 0\\ \vdots & \ddots & \vdots \\ 0 & 0 & X_{B} \end{array}\right]\\ V=\left[\begin{array}{ccc} Z_{A}K\sigma_{u_{A}}^{2}Z_{A}^{\prime }+I\sigma_{\varepsilon_{A}}^{2} & \cdots & Z_{A}K\sigma_{u_{A,B}}Z_{B}^{\prime }+I\sigma_{\varepsilon_{A,B}}^{2}\\ \vdots & \ddots & \vdots \\ Z_{A}K\sigma_{u_{A,B}}Z_{\text{B}}^{\prime }+\text{I}\sigma_{\varepsilon_{A,B}} & \cdots & Z_{\text{B}}\text{K}\sigma_{u_{B}}^{2}Z_{\text{B}}^{\prime }+\text{I}\sigma_{\varepsilon_{B}}^{2} \end{array}\right] \end{array}$$

where **K** is the additive genetic relationship matrix (n × n; the number of lines) calculated by $K=\frac{WW^{\prime }}{2\sum_{k}p_{k}q_{k}}$ using the n × m-centered genotype matrix W, with p and q being allele one and two for the *k*-th genotype and was implemented using the “a.mat” function in R for the *k*-th random effect (*u* = 1,…,k); and **I** is an identity matrix (n × n; n = number of lines) for the residual term. The terms $\sigma_{uk_{i}}^{2}$ and $\sigma_{\varepsilon_{i}}^{2}$ denote the genetic and residual variance of the trait *i*, respectively, and σ_*uk*_*AB*__ and σ_ε_*AB*__ are the genetic and residual covariance between traits A and B. Genetic correlations (*r*_*G*_) between seedling emergence and coleoptile length were then calculated using the function “cov2cor” in the R package sommer (Covarrubias-Pazaran, 2018; R Core Team, 2018).


(10) $$r_{G}=\frac{\sigma_{uk_{AB}}}{\sqrt{\sigma_{uk_{A}}^{2}*\sigma_{uk_{B}}^{2}}}$$

### Genotypic Data

Lines were genotyped by using genotyping-by-sequencing (GBS; Elshire et al., 2011) through the North Carolina State Genomics Sciences Laboratory in Raleigh, NC, United States, using the restriction enzymes *Msp*I and *Pst*I (Poland et al., 2012). Genomic DNA was isolated from seedlings in the one- to three-leaf stage using Qiagen BioSprint 96 Plant kits and the Qiagen BioSprint 96 workstation (Qiagen, Germantown, MD, United States). DNA libraries were prepared following the protocol of DNA digestion with *Pst*I and *Msp*I restriction enzymes (Poland et al., 2012). Genotyping by sequencing (GBS; Elshire et al., 2011) was conducted at North Carolina State University Genomic Sciences Laboratory with either an Illumina HiSeq 2500 or a NovaSeq 6000. DNA library barcode adapters, DNA library analysis, and sequence single-nucleotide polymorphism (SNP) calling were provided by the USDA Eastern Regional Small Grains Genotyping Laboratory (Raleigh, NC, United States). Sequences were aligned to the Chinese Spring International Wheat Genome Sequencing Consortium (IWGSC) RefSeq v1.0 (Appels et al., 2018) using the Burrows-Wheeler Aligner (BWA) 0.7.17 (Li and Durbin, 2009). GBS SNP markers were called using the TASSEL-GBS v2 SNP calling pipeline in Tassel v5 (Bradbury et al., 2007; Glaubitz et al., 2014). Since all the lines in both populations were considered inbred lines, genetic markers with heterozygote calls over 10% were filtered out. Genetic markers common across both populations were combined for quality control, and markers with more than 20% missing data, minor allele frequency of less than 5%, and those that were monomorphic were removed. The markers were then imputed using Beagle version 5.0 and filtered once more for markers under a 5% minor allele frequency (Browning et al., 2018). A total of 40,368 SNP markers remained.

All winter wheat lines in the DP were genotyped with Kompetitive Allele Specific PCR (KASP^®^) assays in the WSU Winter Wheat Breeding Laboratory using allele-specific SNP markers for semi-dwarf causing mutant alleles *Rht-B1b* and *Rht-D1b* previously reported in Grogan et al. (2016) and Rasheed et al. (2016). The KASP assays were performed using PACE™ Genotyping Master Mix (3CR Bioscience, Harlow, United Kingdom) following the instructions of the manufacturer, and endpoint genotyping was conducted from fluorescence using a Lightcycler 480 Instrument II (Roche, Indianapolis, IN, United States). The Rht markers were coded as lines with *Rht-B1b* (1), *Rht-D1b* (2), *Rht-B1b* heterozygous (3), *Rht-D1b* heterozygous (4), *Rht-D1b* with a heterozygous *Rht-B1b* (5), *Rht-B1b* with a heterozygous *Rht-D1b* (6), and both *Rht-B1b* and *Rht-D1b* heterozygous (7).

Linkage disequilibrium between marker pairs was evaluated using JMP Genomics v.9.0 (SAS Institute, Inc, 2011). Significant marker pairs in the same chromosome were considered in LD at a *p*-value < 0.05. Population structure within both populations was analyzed using PC analysis biplots and *k*-means clustering using the markers in the DP and BL populations individually using the function “prcomp” and “cluster” in R, respectively (R Core Team, 2018).

### Genome-Wide Association Models

To dissect the genetic architecture of a complex trait (seedling emergence), the ST-GWAS models were implemented using the Genome Association and Prediction Integrated Tool (GAPIT; Liu et al., 2016; Tang et al., 2016; Huang et al., 2019). Both the ST-GWAS and MT-GWAS models were implemented with three PCs fitted as fixed effects. Three PCs were used based on BIC values using model selection in GAPIT (Tang et al., 2016). The GWAS models were conducted on seedling emergence using the adjusted means mentioned previously and on the BLUPs for coleoptile length. The DP was used as the primary population for genetic dissection, and the BL population was used as the validating population. Three ST-GWAS models were used for comparison. The single-locus ST-GWAS model used was an MLM, and the multi-locus models were BLINK and FarmCPU. Within the DP, we compared each model within each year combination without covariates, then with the Rht markers as covariates, coleoptile length BLUPs, and both Rht markers and coleoptile length as covariates. This resulted in 28 datasets for ST-GWAS for seedling emergence in the DP. The GWAS models were then conducted within the BL without covariates to validate the significant markers. In addition, the ST-GWAS models were used to dissect coleoptile length within the DP for further validation to determine whether the significant markers affected coleoptile length.

Additionally, MTMM was implemented using the “sommer” package for MT-GWAS to identify pleiotropic interactions between seedling emergence and coleoptile length within the DP using the multivariate models 8 and 9. The MT-GWAS model was then implemented in the package “sommer” to obtain marker effects using the inverse of the phenotypic variance matrix (V) and is a generalized linear model of the form:


(11) $$\text{b}=\left({\text{X}}^{{}^{\prime }}\text{V}-\text{X}\right){\text{X}}^{{}^{\prime }}\text{V}-\text{y}$$

(12) $$with\text{X}={\text{ZM}}_{i}$$

where **b** is the marker effect (1 × 2), y is the multivariate response variable (1 × 2), **V-** is the inverse of the phenotypic variance matrix V (2 × 2), **Z** is the incidence matrix for the random effect to perform the GWAS, and M_*i*_ is the *i*-th column of the marker matrix. Furthermore, we implemented three additional *F*-tests for joint analysis using the scripts proposed in Korte et al. (2012). The full (FULL) model, which includes the effect of the marker genotype and its interaction, was tested against a null model and identified both loci with common and interaction effects. The interaction model (IE) for identifying the interaction effects between the traits tested the full model against a genetic model. Finally, we identified common (COM) genetic effects and tested the genetic model against a null model.

Significant associations were based on a Benjamini–Hochberg false discovery rate (FDR) threshold (Benjamini and Hochberg, 1995). The phenotypic variation and effect explained by each significant marker were calculated by conducting stepwise linear regression between phenotypic and genotypic data and calculating the difference between the effects and variation when a single significant marker was added to the null model in R (R Core Team, 2018; Lozada et al., 2019). Significant markers were deemed consistent when they were significant in at least 2 years or in both populations and were used to display reliability of the effect and significance of the markers. We then tested the additive effect of pyramiding the consistent markers identified across populations and the consistent markers identified in multiple years from the DP in each population individually. Manhattan plots were created using the “ggplot2” package, and QQ plots were plotted using the package “CMplots” in R (R Core Team, 2018; Yin et al., 2021).

## Results

### Phenotypic Data

Heritability for seedling emergence was moderately high in the BLs and a few years in the DP, whereas the heritability decreased in combined years. The highest heritability in a single trial was 0.88 in the DP in 2018, and the BLs had a heritability of 0.77 (Table 2). The trials with larger negative adjusted mean values have a larger SD, which indicates a wider range of seedling emergence and increased environmental pressure and phenotypic variation for selection purposes (Supplementary Figure 1). Heritability for coleoptile length was very high in the DP (0.89) (Supplementary Table 1).

**TABLE 2:** Cullis heritability and trial statistics for adjusted means for deep-sowing seedling emergence in individual and combined trials for the diversity panel (DP) population and breeding line (BL) population phenotyped from 2015 to 2019 and 2015, respectively.

Population	Trial	Year	Heritability	CV^a^	Max^b^ (%)	Mean (%)	Min^c^ (%)	SD^d^
DP	DP	2015	0.75	78.91	139	48	−43	38
DP	DP	2017	0.70	24.18	118	87	−26	21
DP	DP	2018	0.88	45.46	118	62	−43	28
DP	DP	2019	0.68	106	135	43	−45	45
DP	DP	2015–2017	0.63	34.53	126	68	−17	23
DP	DP	2015–2018	0.61	28	110	66	2	18
DP	DP	2015–2019	0.64	31.58	101	60	1	19
BL	F_3:5_	2015	0.77	53.55	121	61	−59	33

*^a^CV: coefficient of variation.*

*^b^Max: maximum.*

*^c^Min: minimum.*

*^d^SD: standard deviation.*

**FIGURE 1:**
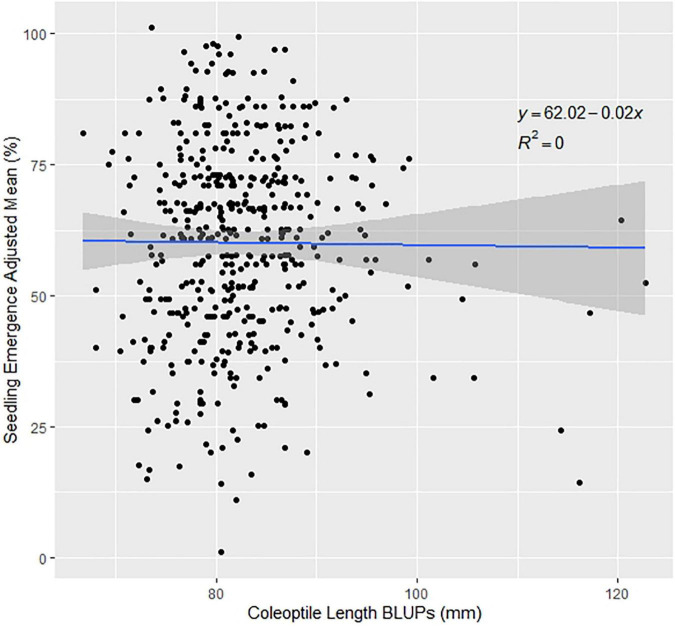
Effect of coleoptile length on seedling emergence in the diversity panel varieties across the combined years of 2015–2019.

Since the variation for seedling emergence depends on environmental effects, it is important to examine the variance components of trials. ANOVAs were conducted using models 3 and 4. Genetic variances were significant for the DP in 2015, 2017, and for all the combined trials (Supplementary Table 2). The environmental effect was not significant in any of the combined trials but had a very large variance for the combined trials. However, for the nested genotype to environment and nested block to environment had large significant variances displaying significant genotype-by-environment interaction (GEI) over the combined trials of 2015–2018 and 2015–2019 (Supplementary Table 2). Furthermore, phenotypic correlations allow for us to compare the results in our GWAS models. The DP trials are significantly positively correlated to each other except for three scenarios: DP 2015 to DP 2018, DP 2017 to DP 2018, and DP 2017 to DP 2019 (Supplementary Table 3). The BL F_3:5_ trial in 2015 was significantly correlated to DP 2017. The genetic correlations between the DP seedling emergence to coleoptile length showed moderate to large correlations in and across years (Table 3). The highest genetic correlation was found in DP 2019, with a correlation of 0.66. However, this was not the case for the phenotypic correlations. The phenotypic correlations in all years were near zero between seedling emergence and coleoptile length (Table 3 and Figure 1).

**TABLE 3:** Genetic and phenotypic correlations between adjusted means for coleoptile length and deep-sowing seedling emergence in individual and combined trials for the DP population phenotyped from 2015 to 2019.

Year	DP 2015	DP 2017	DP 2018	DP 2019	DP 2015–2017	DP 2015–2018	DP 2015–2019
Genetic correlations (*R*^2^)	0.42	−0.40	0.38	0.66	0.19	0.32	0.52
Phenotypic correlations (*R*^2^)	0.00	−0.01	−0.06	0.03	0.00	−0.04	−0.01

### Genotypic Data

**FIGURE 2:**
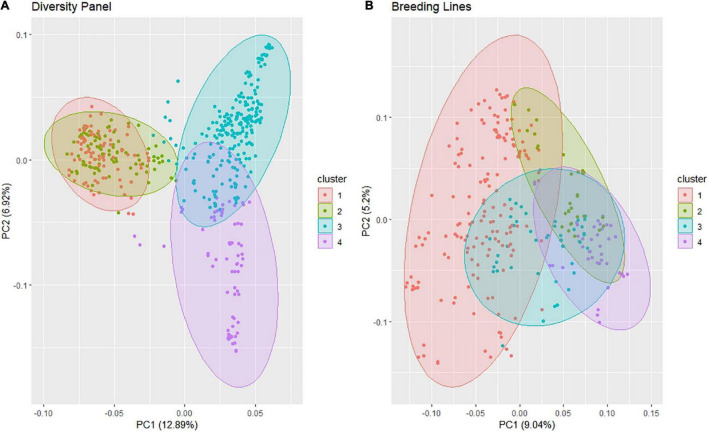
Principal component (PC) biplot of single-nucleotide polymorphism (SNP) genotyping-by-sequencing (GBS) markers and *k*-means clustering from the **(A)** diversity panel and **(B)** breeding line population consisting of the F_3:5_ trial.

The PC biplot using the SNP markers for the DP displayed four clusters according to the elbow method with much overlap between clusters 1 and 2, and a slight overlap between clusters 3 and 4 (Figure 2A). PC1 explained 12.9% of the variation, and PC2 explained 6.9% of the variation. However, most of the lines clustered within a single cluster in the BL population even though the elbow method revealed four clusters using *k*-means, and the biplot explained less variation with 9 and 5.2% for PC1 and PC2, respectively (Figure 2B). We can visually see the larger genetic variation in the DP than in the BLs; therefore, it is important to be accounted for in our GWAS models.

**FIGURE 3:**
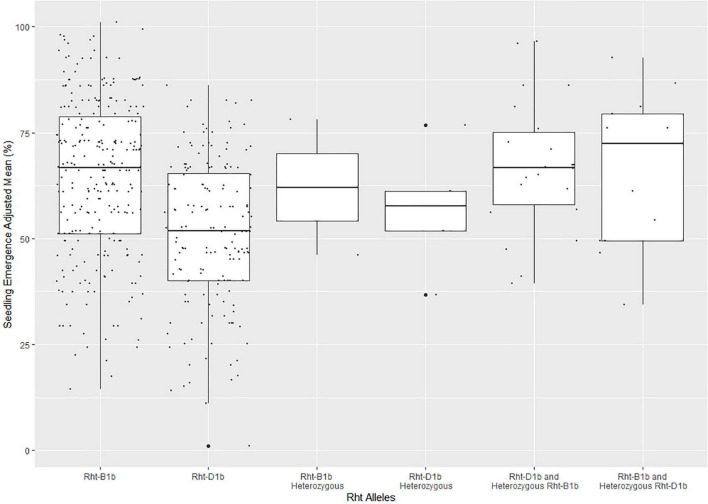
Effect of Rht allele frequency on seedling emergence in the diversity panel varieties across the combined years of 2015–2019. The Rht markers were coded as lines with Rht-B1b, Rht-D1b, Rht-B1b heterozygous, Rht-D1b heterozygous, Rht-D1b with a heterozygous Rht-B1b, Rht-B1b with a heterozygous Rht-D1b, and both Rht-B1b and Rht-D1b heterozygous.

The frequency of the Rht alleles in the DP population can be seen in Supplementary Table 3. The majority of the lines in the DP had either *Rht-B1b* (0.564) or *Rht-D1b* (0.347), with *Rht-B1b* conveying higher mean seedling emergence than *Rht-D1b* (Figure 3). Furthermore, Figure 3 displays outliers for seedling emergence for lines with certain Rht alleles. The outliers were not removed because the cause of poor seedling emergence was indeterminate because of the complexity between phenotypic variation and genotypic effect.

### Single-Trait Genome-Wide Association Studies

To investigate the pleiotropic effects of significant markers, the ST-GWAS was conducted on coleoptile length within the DP. Genome-wide association for coleoptile length displayed three unique markers using MLM, BLINK, and FarmCPU (Table 4). BLINK and MLM both identified a marker, *S1A_14084576*, on chromosome 1A. Marker *S1A_14084576* conveyed the largest *R*^2^ value of any significant marker with a value of 7% and an effect of 14.5 mm. The marker with the next largest *R*^2^ of 0.05 was *S6A_543395015*. It was identified by both BLINK and FarmCPU on chromosome 6A, and it also conveyed the largest effect with 34.4 mm. A third marker was identified only by FarmCPU, *S2A_765677087*, and was located on chromosome 2A. However, *S2A_765677087* had a rather small effect and *R*^2^ of 3.2 mm and 0.04, respectively. The three markers significantly associated with coleoptile length were not identified in any population, year, or model for seedling emergence (Supplementary File 1).

**TABLE 4:** Significant markers for coleoptile length in a Pacific Northwest winter wheat diversity panel using Bayesian information and linkage-disequilibrium iteratively nested keyway (BLINK), fixed and random model circulating probability unification (FarmCPU), and mixed linear (ML) genome-wide association study (GWAS) models.

Marker	Position*^a^*	Alleles*^b^*	Chr*^a^*	Model	*p*-Value	MAF*^c^*	Effect (mm)*^d^*	*R* ^2e^
S1A_14084576	14084576	T/A	1A	BLINK	1.82E−11	0.01	14.52	0.07
				MLM	4.78E−08	0.01	14.52	0.07
S2A_765677087	7.66E+08	G/T	2A	FarmCPU	4.22E−09	0.14	3.23	0.04
S6A_543395015	5.43E+08	T/G	6A	BLINK	4.70E−08	0.00	34.44	0.05
				FarmCPU	6.32E−14	0.00	34.44	0.05

*^a^Chromosomes and positions of markers were determined according to IWGSC RefSeq v.1.1.*

*^b^Allele: favorable allele is underlined.*

*^c^MAF: minor allele frequency.*

*^d^Effect: increase of seedling emergence (%) with the inclusion of favorable allele of associated marker.*

*^e^R^2^: phenotypic variance explained by associated marker.*

There were 107 unique markers over all the combinations of ST-GWAS for seedling emergence (Supplementary File 1). Of the 107 significant markers, 96 markers were significant in the DP and 15 were significant in the BLs, with four markers significant in both populations. Seventy-five of the markers were significant in at least two combinations over both populations. Seventy-one and five markers were significant in at least two combinations in the DP and BLs, respectively. Significant markers were found on 19 of the 21 chromosomes in the DP, with the majority (34) of the significant markers located on chromosome 5A. In the BLs, 15 unique markers spanned nine chromosomes. All the three models had more significant markers in the DP population than in the BL population. The MLM identified 36 unique markers over all GWAS, three in the BLs, and 23 in the DP. FarmCPU identified 65 unique markers overall, nine in the BLs, and 57 in the DP. BLINK identified 31 unique significant markers overall, eight in the BL, and 23 in the DP. The MLM identified significant markers mainly on chromosome 5A with many neighboring significant markers. In contrast, FarmCPU and BLINK identified the same markers on chromosome 5A and identified more markers on various other chromosomes (Supplementary File 1).

**FIGURE 4:**
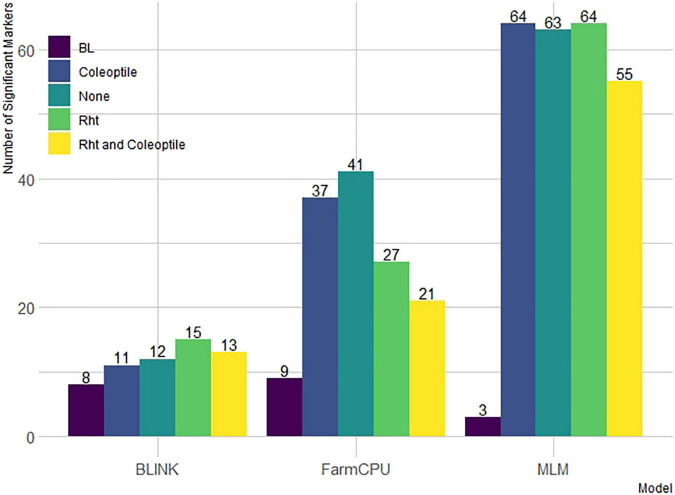
Models compared are Bayesian information and linkage-disequilibrium iteratively nested keyway (BLINK), fixed and random model circulating probability unification (FarmCPU), and mixed-linear model (MLM) with and without Rht and coleoptile length as covariates in the DP and without covariates in the breeding line (BL).

Additionally, there was a differential effect of BLINK and FarmCPU to detect significant markers by including covariates. First, we compared the number of significant markers identified within the DP (Figure 4). For FarmCPU, including both coleoptile length and Rht alleles individually and in combination decreased the number of significant markers compared to FarmCPU without covariates. However, for BLINK, Rht alleles, and the combination of Rht alleles and coleoptile length increased the number of significant markers. Coleoptile length alone decreased the number of significant markers compared to BLINK without covariates. Conversely, the effect of covariates was limited in the MLM. For example, including coleoptile length and Rht alleles as covariates alone resulted in a similar number of significant markers (64 and 63) compared to the MLM without covariates. Including both Rht alleles and coleoptile length did reduce the number of significant markers to 55. Furthermore, a large number of markers in the LD increased the number of significant markers identified by the MLM compared to the other ST-GWAS models.

Second, we compared the effect of including covariates by examining QQ plots. The markers should follow the quantile line in the QQ plot with the exception of a few markers toward the end of the line. A large deviation for a few markers, with the remaining markers on the quantile line, can indicate the power to identify significant markers and fewer false positives. However, a large number of markers deviating from the line can indicate an increase in false positives. For example, in the combined analysis of 2015–2018 and in the BLs in 2015, the MLM was consistent regardless of covariates, which show little effect on the ability to identify significant markers (Supplementary Figure 2). However, this was not the case with BLINK or FarmCPU. Within the DP, the inclusion of Rht or Rht and coleoptile length increased the deviation from the quantile line for the markers at the end of the quantile line more than the models without covariates (Supplementary Figures 3, 4). This displays the advantage of using covariates, such as Rht alleles, within the DP for an increase in detection of significant markers when the trait in question is genetically correlated with another trait. In addition, the QQ plots reveal the difference between the models in the different populations. For both BLINK and FarmCPU, the BL population had the largest deviations compared to the DP GWAS, whereas the opposite was seen in the MLM.

In addition, the environmental impact on seedling emergence had a large effect on the power to identify significant markers. The varying number of significant markers showed the GEI for seedling emergence in different years for the DP. The GWAS models were able to identify the most significant markers in the 2015 trial, with 132 markers (Supplementary Figure 5). However, only a few significant markers were identified in the other individual years, with two in 2017 and five in 2019. Furthermore, combining years and accounting for GEI in our phenotypic adjustments allowed for the GWAS models to increase the ability and power to dissect seedling emergence compared to individual years. All 3-year combinations increased the number of significant markers consistently compared to individual years, with 109 in year combinations 2015–2017 and 105 in 2015–2018. Additionally, there was a decrease in significant markers identified with an increase in year combinations with the combination of all years (2015–2019), identifying only 47 markers (Supplementary Figure 5).

### Multi-Trait Genome-Wide Association Studies

**FIGURE 5:**
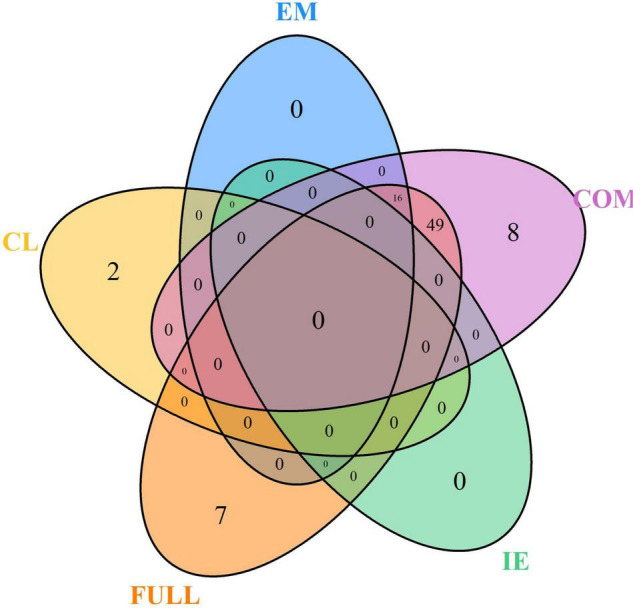
Venn diagram for the number of unique significant markers across the multi-trait genome-wide association studies (MT-GWASs) for seedling emergence (EM), coleoptile length (CL), and the joint analysis for seedling emergence and coleoptile using the full effect (FULL), interaction effect (IE), and common effect (COM) multi-trait mixed models for identifying significant loci controlling deep-sowing seedling emergence and coleoptile length in a Pacific Northwest winter wheat diversity panel (DP) phenotyped across trials from 2015 to 2019 in Lind, WA.

The MT-GWAS models were used to identify pleiotropic loci between seedling emergence and coleoptile length. The MT-GWAS models displayed very similar results for the individual traits compared to the ST-GWAS models, especially in comparison to the single trait results from the MT-GWAS model. However, we used the Bonferroni cut-off with an alpha = 0.05 instead of FDR because of the large deviations and inflation of *p*-values seen on the QQ plots for the joint models (Supplementary Figure 6). Using FDR resulted in 924 unique markers across the majority of chromosomes. In comparison, the Bonferroni cut-off resulted in 82 unique significant markers across 14 chromosomes with the majority of the large effect alleles on chromosome 5A (Supplementary File 2). In comparing the results for MT-GWAS FULL, IE, and COM *F*-tests to the single trait results from the MT-GWAS, we see no significant markers for coleoptile length for any other model (Figure 5). The significant markers for coleoptile length were the same two significant markers on chromosomes 1A and 6A identified in the ST-GWAS models. Additionally, the significant markers for seedling emergence were located on chromosomes 2B (1) and 5A (15).

Furthermore, there were no significant markers in the IE, indicating no contradictory effect markers between seedling emergence and coleoptile length. The lack of significant IE markers is why the FULL and COM models are very similar. The COM model also identified significant markers in every year in the DP compared to the single trait results for seedling emergence, which did not identify significant markers in the 2017 through 2019 individual years (Supplementary File 2). Additionally, there were 64 significant markers in the COM model, with 16 significant markers also found significant for seedling emergence (Figure 5). These markers indicate the potential for confounding effects and the ability to identify them using a joint analysis model compared to the single trait results implemented in the MT-GWAS models.

### Consistent Significant Markers

Because of the lack of identified loci for seedling emergence, the identified markers were compared for significance across years, populations, and correlated traits to insure consistency. No markers were significantly associated for both seedling emergence and coleoptile length in either ST-GWAS or MT-GWAS. Using ST-GWAS models within the DP, 23 out of 107 unique markers were found significant in more than 1 year. BLINK only identified one marker, *S5A_522153944*, across years. FarmCPU identified six markers across years on chromosomes 2A (2), 2B (1), and 5A (3). The MLM identified 16 markers across years, with all but one on chromosome 5A, and with the other marker located on chromosome 5B. For these markers in the DP, only *S5A_522153944* was identified by all three models.

**FIGURE 6:**
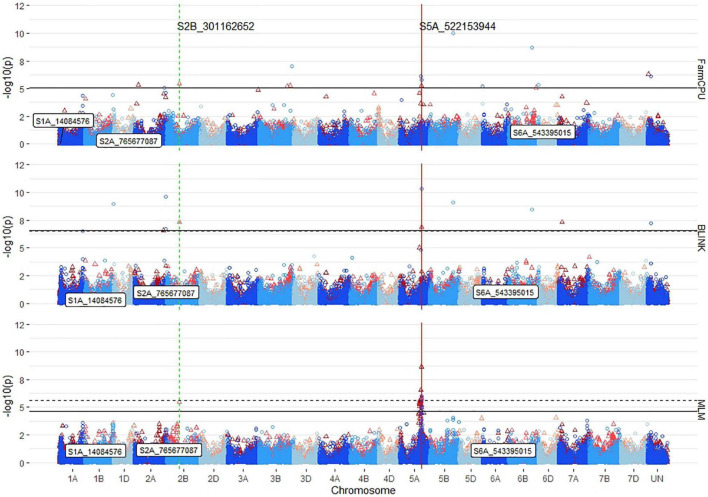
Stacked Manhattan plots for GWASs using MLM, FarmCPU, and BLINK for identifying significant loci controlling deep-sowing seedling emergence in a Pacific Northwest winter wheat diversity panel (DP) phenotyped across trials from 2015 to 2018 and a BL trial phenotyped in 2015 in Lind, WA. Red triangles display GWAS results in the DP, and blue circles identify GWAS results in the BL. Significant markers using a false discovery rate (FDR) cut-off with an alpha = 0.05 are placed above the solid black line for the DP and the dashed black line for the BL. Significant markers across both populations are identified with a vertical red solid line identifying their positions. Markers enclosed in a white text box display significant markers identified in GWASs for coleoptile length.

For the number of significant markers across chromosomes, QQ plots, covariate effect, and populations, FarmCPU displayed the ability to identify both the consistent large effect and small effect markers with fewer false positives because of LD on chromosome 5A (Figure 6). In comparison, the MLM consistently identified 13 markers on 5A with *R*^2^ LD values above 0.8. The two largest effect consistent markers in the DP, *S5A_522153944*, and *S5A_522153953*, were found on chromosome 5A and improved seedling emergence by 30%. These two markers had a maximum *R*^2^ of 10% in the DP. Other consistent markers identified across years in the DP were located on chromosomes 2A (2), 2B (3), 5A (15), 5B (1), and 7A (1). Overall, the consistent markers across years accounted for 30% of the combined 2015–2019 DP variation. The GWAS on the BL population displayed more consistent results than in the DP, and only identified consistent markers on chromosome 5A. There were four significant consistent markers identified in the BLs (Table 5). The four consistent markers were all found in the DP, and had effect ranges of 17–30%, with *R*^2^ values of 12–13%. The consistent markers in the BL population accounted for 18% of the total variation for seedling emergence, displaying more consistent dissection of the complex trait compared to the DP.

**TABLE 5:** Consistent significant markers across both the diversity panel and breeding lines for controlling deep-sowing seedling emergence in a Pacific Northwest winter wheat breeding trial using BLINK, FarmCPU, and ML genome-wide association studies (GWAS) models using covariates for coleoptile length and Rht alleles.

Marker	Position^a^	Alleles^b^	Chr^a^	Pop^c^	Year	Model	Covariate^d^	*p*-Value	MAF^e^	*R* ^2f^	Effect (%)^g^
S5A_514260464	5.14E+08	C/T	5A	BL	15	FarmCPU	N	7.86E-07	0.23	0.13	17
				DP	15	MLM	N, R, C, B	1.81E-09	0.11	0.08	19
				DP	15–17	MLM	N, R, C, B	6.69E-07	0.11	0.05	9
				DP	15–18	MLM	N, R, C, B	6.48E-06	0.11	0.02	5
S5A_522153944	5.22E+08	C/T	5A	DP	15	BLINK	N, C	1.58E-11	0.04	0.09	29
				DP	15–17	BLINK	N, C, B	3.27E-12	0.04	0.08	17
				DP	15–18	BLINK	N, R, C	1.83E-06	0.04	0.10	15
				DP	15–19	BLINK	N, R, C, B	1.82E-09	0.04	0.06	12
				BL	15	FarmCPU	N	1.69E-06	0.04	0.12	30
				DP	15	FarmCPU	N, C	2.44E-06	0.04	0.09	29
				DP	15–18	FarmCPU	N, R	5.13E-06	0.04	0.10	15
				DP	15–19	FarmCPU	R, C, B	6.15E-06	0.04	0.06	12
				BL	15	MLM	N	9.29E-07	0.04	0.12	30
				DP	15	MLM	N, R, C, B	3.62E-09	0.04	0.09	29
				DP	15–17	MLM	N, R, C, B	7.77E-09	0.04	0.08	17
				DP	15–18	MLM	N, R, C, B	2.67E-09	0.04	0.10	15
S5A_522153953	5.22E+08	A/G	5A	DP	15–17	BLINK	R	6.88E-13	0.04	0.08	17
				DP	15	FarmCPU	B	1.03E-07	0.04	0.09	29
				DP	15–18	FarmCPU	B	4.54E-07	0.04	0.10	15
				BL	15	MLM	N	9.29E-07	0.04	0.12	30
				DP	15	MLM	N, R, C, B	3.62E-09	0.04	0.09	29
				DP	15–17	MLM	N, R, C, B	7.77E-09	0.04	0.08	17
				DP	15–18	MLM	N, R, C, B	2.67E-09	0.04	0.10	15
S5A_523025549	5.23E+08	A/G	5A	BL	15	BLINK	N	4.56E-11	0.04	0.12	31
				BL	15	MLM	N	2.57E-06	0.04	0.12	31
				DP	15	MLM	N, R, C, B	1.03E-06	0.05	0.06	23
				DP	15–17	MLM	N, R, C, B	1.15E-05	0.05	0.05	13
				DP	15–18	MLM	N, R, C, B	1.33E-06	0.05	0.07	12

*^a^Chromosomes and positions of markers were determined according to IWGSC RefSeq v.1.1.*

*^b^Allele: favorable allele is underlined.*

*^c^Pop: BL, breeding lines; DP, diversity panel.*

*^d^Covariate: N, none; R, reduced height alleles; C, BLUPs for coleoptile length; B, both reduced height and coleoptile length.*

*^e^MAF: minor allele frequency.*

*^f^R^2^: phenotypic variance explained by associated marker.*

*^g^Effect: increase of seedling emergence (%) with the inclusion of favorable allele of associated marker.*

Out of the 107 unique significant markers, only four were identified in both populations. Furthermore, only *S5A_522153944*, *S5A_522153953*, and *S5A_523025549* were identified across both populations with the same model (Table 5). The MLM identified all three of these markers in both populations. Whereas BLINK did not identify any markers in both populations, FarmCPU only identified S5A_522153944. The effect of covariates on identifying the consistent markers was inconsistent in the DP. The MLM displayed no response to the inclusion of covariates (Supplementary Figure 7). However, FarmCPU identified the consistent marker *S5A_522153944* on chromosome 5A using the Rht alleles, coleoptile length, and Rht alleles with coleoptile length in combination as covariates (Supplementary Figure 8). Furthermore, BLINK was able to detect *S5A_522153944* with and without covariates but was not able to identify it in the BL population (Supplementary Figure 9).

**FIGURE 7:**
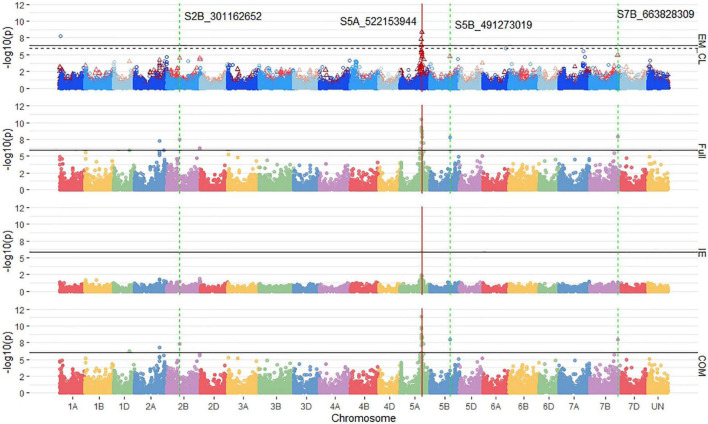
Stacked Manhattan plots for multi-trait genome-wide association studies (MT-GWASs) using seedling and emergence overlapped (EM_CL), the full effect (Full), interaction effect (IE), and common effect (COM) multi-trait mixed models for identifying significant loci controlling deep-sowing seedling emergence and coleoptile length in a Pacific Northwest winter wheat diversity panel (DP) phenotyped across trials from 2015 to 2017 in Lind, WA. For the overlapped plot, EM_CL, red triangles display GWAS results for seedling emergence, and blue circles identify GWAS results for coleoptile length. Significant markers using a Bonferroni cut-off with an alpha = 0.05 are placed above the solid black line for seedling emergence and the dashed black line for coleoptile length. Significant markers across three models are highlighted with a solid red vertical line, and significant markers across two models are highlighted with a dashed green vertical line identifying their positions.

The MT-GWAS models displayed very similar results for identifying consistent markers across years for both the single trait and joint models as the ST-GWAS models. Using single trait MT-GWAS models, consistent markers for seedling emergence were located on 2B and 5A, whereas for coleoptile length, they were identified on chromosomes 1A and 6A. These results are similar to those found using ST-GWAS models. The COM MT-GWAS model identified 19 consistent markers on chromosome 5A, including the large effect marker *S5A_522153944* (Figure 7). In addition, the COM model identified consistent markers on chromosomes 2B (1), 5B (1), and 7B (3). The 2B and 5B markers were *S2B_301162652* and *S5B_491273019.* The 7B markers are all completely linked with *R*^2^ values of 1, with the marker *S7B_663828309* having the largest effect. The 7B markers were the only consistent markers in MT-GWAS not found consistent in the ST-GWAS models.

### Effect of Combining Favorable Alleles

The frequencies of the favorable allele for consistent markers across populations and the consistent markers across years for seedling emergence are important for understanding the selection and makeup of the populations. The consistent markers with LD above 0.8 *R*^2^ were binned together with the marker, and the largest effect marker was identified to represent the bin. This resulted in three bins for the markers identified across populations remaining and 12 bins for the markers identified across years remaining (Supplementary Table 5). In addition, the significant marker on 7B identified in the COM MT-GWAS model was included with the markers consistent across years. These bins were used to display the additive effect for seedling emergence. Most of the markers had high favorable allele frequencies (∼90%) in both the DP and BLs (Supplementary Table 5). This shows that these markers have been indirectly selected in both populations.

**FIGURE 8:**
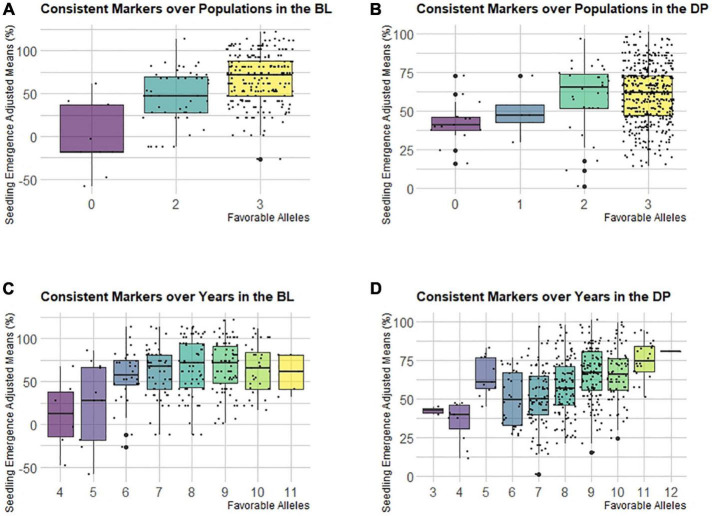
Effect of pyramiding favorable alleles for seedling emergence for the consistent markers identified in both populations **(A,B)**, and consistent markers identified across years in the DP **(C,D)** across both the **(A,C)** diversity panel (DP), and **(B,D)** BL populations.

Even though the consistent markers across populations and years had high favorable allele frequency in the populations, the differences were shown when we combined them and compared the cumulative effect of favorable alleles on seedling emergence (Figures 8A–D). For the consistent markers across populations, the majority of lines in both populations had favorable alleles for all the three bins, which were all located on chromosome 5A. Both populations showed an additive effect, but in the DP, the lines with all three bins had a lower mean compared to the lines with just two bins (Figure 8B). However, in the BLs, there was an increasing trend in emergence with the accumulation of favorable alleles (Figure 8A). The consistent markers across the populations only accounted for 8% of the total variation in the DP and 19% in the BLs. For consistent markers across years, the DP showed an additive effect for all 12 bins, whereas the BLs showed a diminishing return, signifying some of the bins may not have a large effect within the BL population (Figures 8C,D). For the DP and BLs, the majority of lines had either eight or nine favorable alleles. The consistent markers across years accounted for only 23% of the variation in the BLs and 31% in the DP.

## Discussion

### Complex Traits and Seedling Emergence

Complex traits are quantitative in nature and are affected by many small-effect QTLs (Holland, 2007). The challenges that impede the understanding of complex traits are the inability to statistically detect and map minor effect QTLs, accurately understand GEI, and account for pleiotropic effects (Luo et al., 2017). This study attempted to characterize one such trait, seedling emergence for deep-sown winter wheat, to be used as a model for other complex traits.

Our study found no significant associations between seedling emergence and coleoptile length. In previous literature, major QTLs for coleoptile length was reported on chromosomes 4BS, 4BL, and 5AL in wheat (Rebetzke et al., 2001). The QTLs on 4B were reported on either side of the *Rht-B1* gene. This study was further enhanced by a subsequent QTL analysis that resolved the two 4B QTLs directly to the *Rht-B1* locus (Rebetzke et al., 2007). Our study found no significant markers on chromosome 4B for coleoptile length or seedling emergence. Two small effect markers were found on chromosome 4D using the joint COM and FULL MT-GWAS models but were not consistent across years. The major consistent markers validated across populations for seedling emergence in our study were identified on chromosome 5A. In the follow-up study by Rebetzke et al. (2007), QTLs for coleoptile length on chromosome 5A were identified but were not repeatable across populations. In the same study, Rebetzke et al. (2007) identified small-effect QTLs for coleoptile length on wheat chromosomes 1A, 2B, 2D, 3A, 3B, 5A, and 6A. In our study, significant markers for coleoptile length were found on chromosomes 1A, 2A, and 6B and only in the DP, but none were also significant for seedling emergence. Therefore, we conclude that the GWAS models tested are not selecting major markers for coleoptile length, and that the seedling emergence we are dissecting is indeed due to other factors affecting seedling emergence, similar to what was indicated in Mohan et al. (2013).

Our study demonstrated that chromosome 5A is associated with seedling emergence, with four large effect markers identified consistently across both years in the DP and validated in the BLs. Therefore, a major effect of QTL for seedling emergence, with an increase of up to 30%, may be present in that chromosomal location. According to Bernardo (2014), a major marker accounts for >10% of the phenotypic variation. Since this 5A locus is associated with seedling emergence and not with coleoptile length, other associated traits are improving seedling emergence. These associated traits may be fast emergence, the ability to germinate and/or grow under moisture stress, or other unknown mechanisms. Since the variation for emergence in the DP and BLs was due to moisture stress, the 5A locus may affect both fast emergence and the ability to germinate and grow under low moisture. The identification of a few large effects and many small effects significant markers confirms that seedling emergence is a complex trait controlled by multiple pleiotropic factors other than coleoptile length.

### Genome-Wide Association Models

Genetic mapping through association studies has been performed to dissect the genetic architecture of various traits in wheat (Adhikari et al., 2012; Lozada et al., 2017, 2019; Bajgain et al., 2019; Lozada and Carter, 2020). However, GWAS has not been implemented for deep-sowing seedling emergence in winter wheat but has been conducted in rice (Zhao et al., 2018). Even further, few studies have compared ST-GWAS models, and fewer have compared FarmCPU and BLINK (Liu et al., 2016; Huang et al., 2019). By comparing multiple covariates and GWAS models, we attempted to compare the ability of models and covariates to identify significant markers to dissect a trait with unknown genetic architecture. This allowed us to identify large effect markers with up to 30% effect and 10% *R*^2^ values. Therefore, we can conclude that these markers are important for seedling emergence.

The single-locus model used in our study was MLM. The MLM identified significant markers mainly on chromosome 5A and was consistent in identifying large effect markers without the inclusion of covariates. However, the MLM displays low ability in identifying both major markers and other lower effect significant markers in the presence of high levels of LD. Furthermore, while MLMs have been shown to be comparable to FarmCPU (a multi-locus model) for simple traits, the MLMs have been shown to have difficulties with low power and false positives for complex traits (Habier et al., 2011; Liu et al., 2016; Ward et al., 2019). MLMs evaluate the relationship and overall variation of each genetic marker independently; therefore, as the trait increases in complexity and number of pleiotropic effects, the proportion of variation due to a locus decreases (Miao et al., 2019).

Fixed and random model circulating probability unification and multi-locus models provide the best trade-off between power and false positives (Liu et al., 2016; Miao et al., 2019). Multi-locus models increase the proportion of genetic variance using major effect markers as fixed effects and identifying more significant markers (Miao et al., 2019). This is why our multi-locus models were able to dissect the complex trait of seedling emergence accurately and identify both major and minor effect markers. The multi-locus models used in our study were FarmCPU and BLINK and had very similar results in the BL population, but there were fewer significant markers discovered by BLINK in the DP. The difference between BLINK and FarmCPU is that Bayesian Information Criteria in a fixed-effects model replaces REML in the random-effects model, and LD information is used to replace the bin method implemented in FarmCPU. Therefore, since the LD is high among significant markers on the same chromosome in both populations, the amount of markers grouped together may be different, which reduces the ability to identify significant markers on 5A across populations. Apart from the markers on 5A that the MLM also found, most markers found significant by FarmCPU and BLINK had relatively small effects and *R*^2^ values, indicating the ability of multi-locus models to identify small-effect markers for complex traits.

There was little to no increase in identifying pleiotropic loci using MT-GWAS compared to ST-GWAS models. This may be due to the lack of similar significant markers or pleiotropic loci between seedling emergence and coleoptile length, along with the inflated *p*-values we observed in the joint analyses. We observed such large inflation of *p*-values that we had to use the more stringent Bonferroni cut-off as the threshold. The MT-GWAS model we used implemented a single-locus model and, therefore, suffered limitations in identifying small effect loci similar to the MLM. However, the COM model did identify consistent markers across years on chromosome 7A, whereas the ST-GWAS model only identified a single-year combination. The identification of a significant marker in the COM model not seen in the ST-GWAS, combined with the lack of significant interaction effect loci, confirms the positive genetic correlation seen in the majority of years between seedling emergence and coleoptile length. However, due to the lack of evidence of pleiotropic loci in our other methods, the genetic correlation may be due to the correlation of shared loci such as the Rht alleles.

The lack of increase in ability to identify pleiotropic loci using the MT-GWAS models may also be due to the lack of phenotypic correlation between seedling emergence and coleoptile length (Korte et al., 2012). Even though we see a positive genetic correlation, the phenotypic correlation is near zero, indicating a small genetic effect in comparison to the environmental effect. In this scenario, it has been shown that the MT-GWAS models do not outperform ST-GWAS models and confirm why our MT-GWAS models did not outperform the ST-GWAS models (Korte et al., 2012). However, MT-GWAS can still be used as a complement to ST-GWAS models to identify interactions and pleiotropic loci such as the loci on chromosome 7A.

### Covariates

Covariates are commonly used in GWAS studies to allow for models to differentiate genetic and environmental effects (Tibbs Cortes et al., 2021). For a complex trait, more confounding associations such as correlated traits or pleiotropic effects may account for a portion of seedling emergence variation (Saltz et al., 2017). To account for these correlated traits, we compared the use of covariates for *Rht* alleles and coleoptile length. These correlated traits were used because of previous studies reporting large associations between the traits (Allan et al., 1962; Sunderman, 1964; Chastain et al., 1995; Schillinger et al., 1998; Botwright et al., 2001; Schillinger, 2011).

The Rht and coleoptile length covariates had an effect on identifying significant markers for the multi-locus models but not for the MLM. The multi-locus models required the inclusion of covariates in order to identify the major effect of significant markers on chromosome 5A, but not for identifying the small effect markers. Including covariates or secondary traits can account for potential confounding factors that bias marker effect estimates (Aschard et al., 2015). There were no common significant markers between seedling emergence and coleoptile length, including covariates, that affected the ability of the multi-locus models to identify the large effect markers. This indicates that there may be confounding effects between coleoptile length and seedling emergence and that the relationship between coleoptile length and *Rht* alleles in our populations is no longer linear. Therefore, even though modern varieties are no longer dependent on coleoptile length for improved emergence, it may still confer some confounding effects. This is further confirmed by the results of our MT-GWAS models and moderate genetic correlations. Therefore, we recommend using both multi-locus models and covariates for correlated traits to identify both small and large effect loci.

### Association Mapping Populations and Environments

Closer relationships between genotypes indicate that fewer recombination events have occurred, which preserves marker LD and requires a less genetic variation to be accounted for by the models (Habier et al., 2007; Bernardo, 2020). The DP had a more distinct population structure compared to the BLs, indicating that the BL population is more genetically related. The BL population was purposely selected over generations for deep-sowing seedling emergence in the Washington State University breeding program. Even though these lines have diverse pedigrees, they are based on specific founder lines with good emergence, which may be the reason for the increased relatedness. In contrast, the DP is composed of varieties from various breeding programs in the Pacific Northwest, with the majority of lines not bred specifically for deep-sowing seedling emergence. Even with the differences in population structure, we were able to identify the consistent markers on chromosome 5A in both populations.

In addition to the differences between the composition and genetic relatedness of the populations, the number of environments examined between the two populations differed. The BLs only had emergence data for 2015, whereas the DP, an unselected population for seedling emergence, had trait data for multiple years. The combining of years and accounting for GEI in our phenotypic adjustments of the DP allowed for the GWAS models to increase the power to dissect seedling emergence compared to individual years. Furthermore, the lack of consistency between GWAS models and populations confirms the complexity of seedling emergence and the need for evaluation across multiple environments. Since the environment can create phenotypic variation in seedling emergence, it displays GEI. If a trait displays GEI, it follows that so would the QTLs responsible for the phenotypic expression (Bernardo, 2020). A change in the ranking of QTLs across environments indicates QTL-by-Environment Interaction (QEI), with the detection of QTLs in some environments and not others (Bernardo, 2020). Therefore, QEI can be seen with the differing number of significant markers in individual and combined years.

Furthermore, the difficulty in dissecting seedling emergence within and across years can be seen in the difference between the varying genetic and phenotypic correlations between years and traits. The differences in genetic correlations for seedling emergence and coleoptile length from year to year and the near-zero phenotypic correlations can be explained by the large effect of the environment and the multitude of factors that affect seedling emergence. The phenotypic effect can be partitioned into both genetic and non-genetic effects (Searle, 1961). Since genetic correlation only takes into account the genetic effect, we still see moderate values. The negative genetic correlation in 2017 can be explained by the different factors that affect seedling emergence, such as coleoptile diameter, force, and speed of emergence, and not coleoptile length. The fact that other factors are affecting seedling emergence is further confirmed because of the lack of significant markers for the interaction effect in the MT-GWAS models (Korte et al., 2012). If the negative genetic correlation was due to the significant loci for coleoptile length, we would see significant markers for the interaction effect (Korte et al., 2012). Additionally, the near-zero phenotypic correlations display that coleoptile length, while having moderate genetic correlations, has a much smaller genetic effect than the environmental effect, and therefore has little overall phenotypic effect for seedling emergence. In other terms, when the environment has large control over the expression of the trait but a low correlation, the phenotypic correlation would be expected to be lower than the genetic correlation (Searle, 1961).

### Combining Favorable Alleles

We see an increase in seedling emergence and additive effect of accumulating favorable alleles using the consistent markers identified across years in the DP. In contrast, in the BLs, there was an additive effect of accumulating favorable alleles for the consistent markers identified over populations. The difference between the two sets of markers was that the consistent markers over populations were exclusively located on chromosome 5A, whereas the consistent markers over years were located on various chromosomes. All the consistent markers displayed high frequencies in both populations, which demonstrated the success of accumulating favorable alleles through traditional phenotypic selection.

There was a general additive effect with the accumulation of favorable alleles for both marker sets. The consistent markers identified across years were based on the results of the DP and showed a continual increase in seedling emergence as favorable alleles were accumulated. In the BLs, seedling emergence improved through additivity but only to a point; once the number of favorable alleles was high, no improvement was found with continual accumulation. This may be because the DP is an unselected population, whereas the BLs had previously been selected for improved emergence, and therefore may already have fixed a high number of alleles for emergence. Since there was not a large decrease in seedling emergence with the accumulation of favorable markers, we can presume that seedling emergence is generally additive with the possibility of some interaction or non-additive gene action, as seen in other complex traits (Bonnafous et al., 2018). Pyramiding favorable alleles has shown to be successful for disease resistance traits (Naruoka et al., 2015; Lewien et al., 2018; Lozada et al., 2019) and can be equally successful for abiotic traits such as seedling emergence.

## Conclusion

This study displayed the ability of GWAS to dissect the genetic architecture of a complex trait such as deep-sown seedling emergence. Both the ST-GWAS and MT-GWAS models identified a few large effects and many small effect markers for seedling emergence. Additionally, neither the ST-GWAS nor the MT-GWAS models identified large pleiotropic effect markers between seedling emergence and coleoptile length. The ST-GWAS and MT-GWAS models did not identify the same significant markers for seedling emergence or coleoptile length, and the MT-GWAS models did not identify any interaction effect markers. However, by using multi-locus models in conjunction with covariates for correlated traits, we were able to identify more small effect loci over single-locus models to dissect a complex trait in both the DP and BL populations. Additionally, the DP displayed the necessity for combining years for consistent identification of significant markers for a trait dependent on the environment for phenotypic variation. Furthermore, the MT-GWAS models displayed a lower ability to identify small effect loci over ST-GWAS models for single-trait analysis and inflated *p*-values for joint analysis but still identified the large effect markers on 5A. Therefore, using multi-locus models combined with covariates (such as major genes), breeding programs can uncover the complex nature of traits to help identify candidate genes and the underlying architecture of a trait to make more efficient breeding decisions and selection methods.

## Data Availability Statement

The datasets presented in this study can be found in online repositories. The names of the repository/repositories and accession number(s) can be found below: https://github.com/lfmerrick21/GWAS-Complex-Traits.

## Author Contributions

LM conceptualized the idea, analyzed the data, and drafted the manuscript. AB genotyped the KASP markers, and reviewed and edited the manuscript. ZZ reviewed and edited the manuscript. AC supervised the study, conducted field trials, edited the manuscript, and obtained the funding for the project. All authors contributed to the article and approved the submitted version.

## Conflict of Interest

The authors declare that the research was conducted in the absence of any commercial or financial relationships that could be construed as a potential conflict of interest.

## Publisher’s Note

All claims expressed in this article are solely those of the authors and do not necessarily represent those of their affiliated organizations, or those of the publisher, the editors and the reviewers. Any product that may be evaluated in this article, or claim that may be made by its manufacturer, is not guaranteed or endorsed by the publisher.
